# Accurate and molecular-size-tolerant NMR quantitation of diverse components in solution

**DOI:** 10.1038/srep21742

**Published:** 2016-02-17

**Authors:** Hideyasu Okamura, Hiroshi Nishimura, Takashi Nagata, Takanori Kigawa, Takashi Watanabe, Masato Katahira

**Affiliations:** 1 Institute of Advanced Energy, Kyoto University, Gokasho, Uji, Kyoto 611-0011, Japan; 2 RIKEN Quantitative Biology Center, 1-7-22 Suehiro-cho, Tsurumi-ku, Yokohama, Kanagawa 230-0045, Japan; 3 CREST, JST, 5-3 Yonban-cho, Chiyoda-ku, Tokyo 102-8666, Japan; 4 Research Institute for Sustainable Humanosphere, Kyoto University, Gokasho, Uji, Kyoto 611-0011, Japan; 5 Department of Computational Intelligence and Systems Science, Interdisciplinary Graduate School of Science and Engineering, Tokyo Institute of Technology, 4259 Nagatsuta-cho, Midori-ku, Yokohama, Kanagawa 226-8503, Japan

## Abstract

Determining the amount of each component of interest in a mixture is a fundamental first step in characterizing the nature of the solution and to develop possible means of utilization of its components. Similarly, determining the composition of units in complex polymers, or polymer mixtures, is crucial. Although NMR is recognized as one of the most powerful methods to achieve this and is widely used in many fields, variation in the molecular sizes or the relative mobilities of components skews quantitation due to the size-dependent decay of magnetization. Here, a method to accurately determine the amount of each component by NMR was developed. This method was validated using a solution that contains biomass-related components in which the molecular sizes greatly differ. The method is also tolerant of other factors that skew quantitation such as variation in the one-bond C–H coupling constant. The developed method is the first and only way to reliably overcome the skewed quantitation caused by several different factors to provide basic information on the correct amount of each component in a solution.

The first step to analyse a solution of interest is elucidation of both the molecular structure and the amount of each component involved. This basic information is crucial for understanding the physical, chemical and biological properties of the solution. The information is also required for engineering and industrial utilization of components. Among the many analytical methods available, NMR is one of the most powerful and widely used to obtain quantitative information on components in solution in a native state. For example, 2D ^1^H–^13^C heteronuclear single-quantum correlation (HSQC)^1^, in which peak overlap in 1D spectra is mitigated by the increased dimensionality, is widely used to analyse the various components and polymer compositions of solubilized woody biomass^2^,^3^,^4^,^5^. However, as the authors have been careful to point out, because of (primarily) the non-linearity resulting from HSQC-responses to the vastly different relaxation rates of components in the solution, or to end groups vs. internal units in a polymer, only relative integrals are reportable and not true quantitation. The current methods are therefore really only applicable to comparative analyses between samples. Attempts to improve the quantitation^6^,^7^ are unwieldy or do not address all of the issues. Here we describe a new quantitation method, TAF (tolerant of any factors) quantitation method, in which the calibrated volume integral of a ^1^H–^13^C correlation peak is proportional to the absolute amount.

## Results

### Skewing of the Quantitation with HSQC Caused by Size-dependent Decay of Magnetization

**Figure 1:**
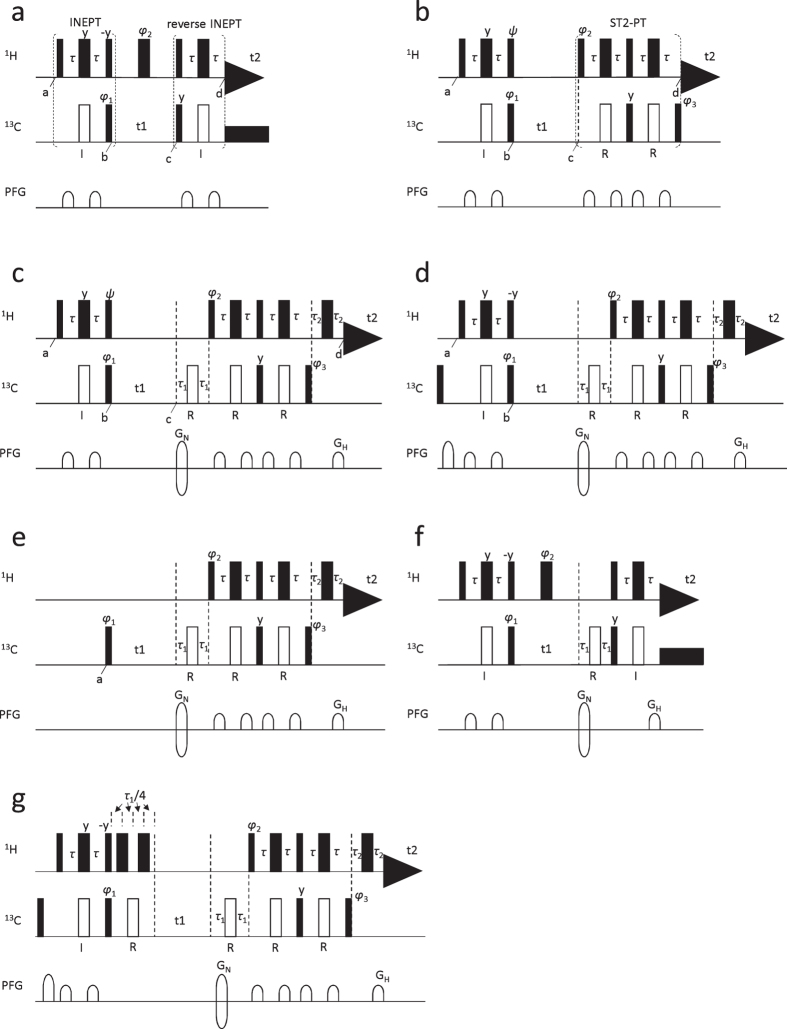
Pulse sequences used. (**a**) ^1^H–^13^C HSQC pulse sequence, (**b**) ^1^H–^13^C TROSY pulse sequence, (**c**) TROSY pulse sequence with coherence-selection, (**d**) TROSY sequence modified from (**c**) to diminish the contribution of *C*_z_, (**e**) TROSY sequence modified from (**c**) to diminish the contribution of *H*_z_, (**f**) HSQC pulse sequence with coherence-selection (HSQC’), and (**g**) TROSY sequence modified from (**d**) to insert a period for extra reduction of magnetization. Narrow and wide bars represent 90° and 180° pulses, respectively. Black and white bars represent rectangular and adiabatic pulses, respectively. I and R indicate pulses for inversion and refocusing. G_N_ and G_H_ are gradients for coherence selection. *τ*, *τ*_1_ and *τ*_2_ were set to 1.563, 1.204 and 1.208 ms, respectively. The repetition delay was set to 10 s. The phase of all pulses is x, unless indicated. Phase cycling and frequency discrimination of each pulse sequence are as follows. (**a**) Phase cycling is *φ*_1_ = y, -y; *φ*_2_ = 2(x), 2(−x); and receiver = 2(x, −x). Frequency discrimination is obtained by States-TPPI phase cycling of *φ*_1_. (**b–e**) Phase cycling for the first FID is *ψ* = −y or y; *φ*_1_ = y, -y, −x, x; *φ*_2_ = y; *φ*_3_ = x; and receiver = x, −x, −y, y. Phase cycling for the second FID is *ψ* = −y or y; *φ*_1_ = −y, y, −x, x; *φ*_2_ = −y; *φ*_3_ = -x; and receiver = x, −x, −y, y. For each *t*_1_ increment, *φ*_1_ and the receiver are inverted. The data are processed as described by Kay *et al.*^12^. (**f**) Phase cycling is *φ*_1_ = *y*, −*y*; *φ*_2_ = 2(*x*), 2(−*x*); and receiver = 2(*x*, −*x*). To obtain a complex interferogram, the gradient G_N_ of a second FID is inverted. For each *t*_1_ increment, *φ*_1_ and the receiver are inverted. The data are processed as described by Kay *et al.*^12^. (**g**) Phase cycling and data processing are the same as (**c**), except for *ψ* and *φ*_1_. For the first FID, *φ*_1_ = −*y*, *y*, −*x*, *x*. For the second FID, *φ*_1_ = *y*, −*y*, −*x*, *x*.

**Figure 2:**
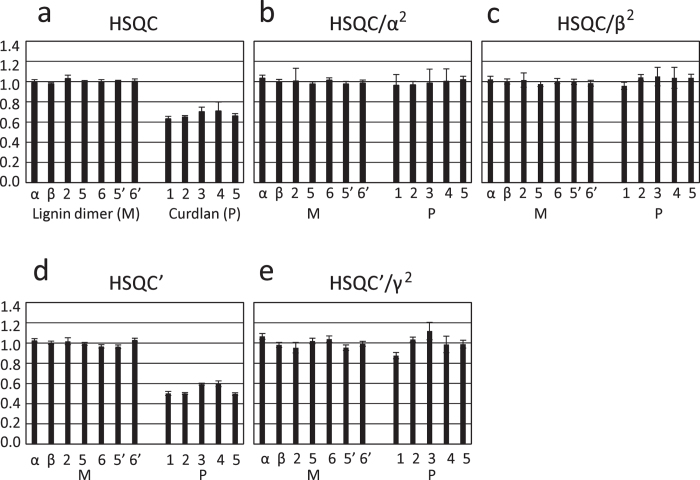
Calibration of volumes of HSQC correlation peaks to correctly determine the amount of each component. The volumes of each correlation peak of the ^1^H–^13^C HSQC spectrum recorded with either the Fig. 1a pulse sequence (**a**) or the Fig. 1f pulse sequence (**d**). The volume of the peak corresponding to each CH moiety was obtained. The volumes are normalized so that the average of the volumes for the peaks of the lignin dimer is 1. Calibrated volumes were calculated and plotted for HSQC/α^2^ (**b**), HSQC/β^2^ (**c**) and HSQC’/γ^2^ (**e**). Error bars represent standard deviations of four independent experimental data sets.

A low molecular weight (320 Da) lignin dimer that includes the most abundant inter-unit linkage in lignin, and curdlan, a high molecular weight (81 kDa average) β-1,3-linked glucan (Supplementary Fig. S1), were dissolved together in DMSO so that the concentration of the lignin dimer was equal to that of the monomer unit of the curdlan on the basis of the area of each peak in the one-dimensional ^1^H spectrum. The HSQC spectrum of this sample was recorded with the Fig. 1a pulse sequence and is shown in Supplementary Fig. S1 with the assignments. The volume of the peak corresponding to each CH moiety was obtained and normalized so that the average of the volume for the peaks of the lignin dimer was 1 (Fig. 2a); the volume of each peak from the lignin dimer was almost the same. The volume of each peak from the curdlan was also almost the same, but much less than 1, the average being 0.671 (Table 1). This is due to the differences in magnetization losses through transverse relaxation during the INEPT and reverse INEPT periods of the HSQC pulse-program. The transverse relaxation rate of the curdlan is much faster than that of the lignin dimer due to the extreme molecular weight difference (81,000 vs. 320)^8^. Therefore, despite the monomer concentrations’ being equal, the loss of magnetization during the periods is much larger for the curdlan units than for the lignin dimer, resulting in a much smaller volume for each curdlan HSQC peak. In this situation, it is impossible to correctly determine the amount of each component in solution from the HSQC spectrum. Development of a method to achieve correct quantitation in such cases has long been sought.

### Analysis of the Skew with Product Operator Formalism and a Hint for Preventing the Skew

The loss of magnetization during the two INEPT periods can be traced using the product operator formalism^9^. At time a in the Fig. 1a pulse sequence, the operator is *H*_z_, and becomes 2*H*_z_*C*_x_ at time b after the INEPT period (where *H* and *C* refer to proton and carbon magnetization). When the transverse relaxation during the INEPT period is taken into account, the virtual magnetization decreases to δ∙2*H*_z_*C*_x_ at time b, where 0 < δ < 1. When *t*_1_ is assumed to be 0 for simplicity, the relevant operator at time c is −δ∙2*H*_z_*C*_x_ and it becomes δ^2^∙*H*_x_ at time d after the reverse INEPT period, where reduction of magnetization due to the transverse relaxation during the reverse INEPT period is also taken into account. It should be noted that the transverse relaxation during the *t*_1_ period affects the width of the correlation peak but does not affect its volume. Therefore, the discussion under the assumption of *t*_1_ = 0 does not lose generality for examining the effect of transverse relaxation on the volume of the correlation peak.

The Fig. 1b pulse sequence is that of transverse relaxation optimized spectroscopy (TROSY)^10^,^11^. Tracing the magnetization for TROSY, *H*_z_ at time a becomes δ∙2*H*_z_*C*_x_ at time b in the same way. δ∙2*H*_z_*C*_x_ can be expressed as δ∙*H*^α^*C*_x_ − δ∙*H*^β^C_x_. When *t*_1_ is 0, the relevant operators at time c are δ∙*H*^α^*C*_x_ − δ∙*H*^β^*C*_x_, and they become δ∙ε∙*H*_y_*C*^β^ + δ∙ε∙*H*_y_*C*^α^ at time d, where ε is such that 0 < ε < 1, the reduction of magnetization due to the transverse relaxation during the ST2-PT (single transition to single transition polarization transfer) period between c and d being taken into account. It should be pointed out that only the second term of δ∙ε∙*H*_y_*C*^β^ + δ∙ε∙*H*_y_*C*^α^, i.e., δ∙ε∙*H*_y_*C*_α_, is detected with a coherence-selection method in an actual experiment, as explained below. In the case of TROSY, *C*_z_ at time a also contributes to the TROSY signal. It becomes −*C*_x_ at time b after the INEPT period. It should be noted that the magnetization does not decrease due to the transverse relaxation during the INEPT period, because the magnetization is always along either the z-axis or −z-axis. −*C*_x_ can be expressed as −*H*^α^*C*_x_–*H*^β^*C*_x_. When *t*_1_ is 0, the relevant operators at time c are −*H*^α^*C*_x_–*H*^β^*C*_x_, and they become −ε∙*H*_y_*C*^β^ + ε∙*H*_y_*C*^α^ at time d, where the reduction during the ST2-PT period is taken into account in the same way. Again, only the second term, ε∙*H*_y_*C*^α^, is detected in an actual experiment. In summary, *H*_z_ at time a becomes δ∙ε∙*H*_y_*C*^α^ at time d, whereas *C*_z_ at time a becomes ε∙*H*_y_*C*^α^. Both contribute to the same TROSY signal. A remarkable point is that H_z_ decreases during both the INEPT and ST2-PT periods, whereas *C*_z_ reduces during just the ST2-PT period. This difference was utilized to develop the new quantification method, as follows.

### The Method for the Correct Quantitation by NMR

Suppose the lower right component of four fine structures of a TROSY correlation peak is detected in an experiment. The intensities of steady-state *H*_z_ and *C*_z_ magnetizations are assumed to be *u* and *v*, respectively. When a phase of the ^1^H pulse labelled with *ψ* in the Fig. 1b pulse sequence is set to −y, henceforth this pulse sequence is designated as pulse b(-y), signals originating from *H*_z_ and *C*_z_ are both added at the TROSY peak. On the other hand, when this phase is set to y, henceforth this pulse sequence is designated as pulse b(y), the latter signal is added with a negative sign^12^. Thus, the volume of the TROSY peak for a b(-y) pulse sequence is proportional to δ∙ε∙*u* + ε∙*v* = ε∙(δ∙*u* + *v*), whereas that for a b(y) pulse sequence is proportional to δ∙ε∙*u*–ε∙*v* = ε∙(δ∙*u*–*v*).

Here we define α for each peak as


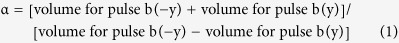


Then, it was deduced that α is equal to δ∙*u*/*v*. Here, *u*/*v* is a constant, so it is concluded that α is proportional to δ. In the case of HSQC, magnetization decreases by a factor of δ^2^ due to transverse relaxation during the INEPT and reverse INEPT periods, as discussed above. Therefore, when the volume of each HSQC peak is divided by α^2^ of each peak, the effect of reduction due to transverse relaxation is cancelled out. Thus, it is expected that the volume of each peak can be quantitatively compared without skew caused by variation in the transverse relaxation rate. Figure 2b clearly indicates that this idea works well in practice. The calculated volumes for curdlan increased and are all close to 1, the average being 0.989 (Table 1). This is exactly what we expect for a mixture in which the concentrations of the lignin dimer and the monomer unit of curdlan are the same.

For a TROSY experiment, only the second term of either δ∙ε∙*H*_y_*C*^β^ + δ∙ε∙*H*_y_*C*^α^ originating from *H*_z_ or −ε∙*H*_y_*C*^β^ + ε∙*H*_y_*C*^α^ originating from *C*_z_ is detected, as mentioned above. In order to reduce the number of phase cycling steps, a coherence-selection method^11^,^12^ is applied to the Fig. 1c pulse sequence by introducing *τ*_1_ - ^13^C 180° pulse – *τ*_1_ and *τ*_2_ - ^1^H 180° pulse – *τ*_2_ units coupled with a gradient after the *t*_1_ period and prior to the *t*_2_ period, respectively. Then, α was calculated for each peak with its volume in the TROSY spectrum obtained via the Fig. 1c pulse sequence, as follows.


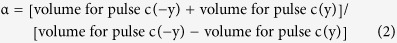


Here, the meaning of c(−y) and c(y) is the same that of b(−y) and b(y). Reduction of magnetization due to transverse relaxation occurs during the times of the two units described above. However, this does not affect α, because the effect is cancelled out due to division in the calculation of α. In fact, Fig. 2b was obtained from the α derived with the Fig. 1c pulse sequence. Thus, a TROSY pulse sequence that incorporates the coherence-selection method can be successfully used to obtain α for the correct quantitation.

Suppose a case in which there are wide distributions of the transverse relaxation rates for a signal of a component in a polymer containing many same components. The magnetization losses during the INEPT and reverse INEPT periods differ depending on the transverse relaxation rate. Concerning a certain signal, our method determines the sum of the magnetization losses caused by various transverse relaxation rates. This information can be used to correct the skew of quantitation for a certain signal in the same way. Thus, our method is valid even if there are wide distributions of the transverse relaxation rates for a signal.

### Alternative Method for the Correct Quantitation

In the method described in the previous section, the intensities of signals originating from *C*_z_ might be slightly distorted during the INEPT period by the longitudinal relaxation after the first ^13^C 180° pulse. In the case of the current sample, the distortion is negligible, less than 1%. But in other cases, the distortion might be problematic. For those cases, we have also developed an alternative method for quantitation utilizing TROSY. For the Fig. 1d pulse sequence, a ^13^C 90° pulse and a gradient are added at the beginning of the Fig. 1c pulse sequence to purge steady-state *C*_z_ magnetization. Thus, for the Fig. 1d pulse sequence, only *H*_z_ is present at time a to contribute to the volume of the TROSY peak. For the Fig. 1e pulse sequence, the INEPT unit of the Fig. 1c pulse sequence is replaced by a ^13^C 90° pulse. Thus, for the Fig. 1e pulse sequence, only *C*_z_ is present at time a to contribute to the volume of the TROSY peak. As discussed above, *H*_z_ at time a becomes −δ∙ε∙*H*_y_*C*^α^ after the ST2-PT period of the TROSY pulse, while *C*_z_ at time a becomes ε∙*H*_y_*C*^α^. Thus, the volume of the TROSY peak for the Fig. 1d pulse sequence is proportional to δ∙ε∙*u*, whereas that for the Fig. 1e pulse sequence is proportional to ε∙*v*. Here, the reduction of magnetization during the [*τ*_1_ - ^13^C 180° pulse - *τ*_1_] and [*τ*_2_ - ^1^H 180° pulse - *τ*_2_] units coupled with a gradient is ignored, because this effect is cancelled out due to division in the following equation to calculate β, as explained above. We define β for each peak as


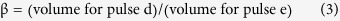


β is equal to δ∙*u*/*v*. Again, *u*/*v* is a constant, so it is concluded that β is proportional to δ. When the volume of each HSQC peak that is affected by the factor δ^2^ is divided by β^2^ of each peak, the effect of reduction due to transverse relaxation is cancelled out, and thus the volume of each peak can be quantitatively compared without skew caused by variation in the transverse relaxation rate. Figure 2c clearly indicates that this alternative idea also works well. The volumes for curdlan are again all close to 1, the average being 1.025 (Table 1).

This alternative method is more rigorous than the one in the previous section. However, the signals originating from *C*_z_ and *H*_z_ are discarded in pulses of Fig. 1de, respectively. Therefore, the alternative method needs more experimental time than the one in the previous section by the factor of √2. So, it is recommended to use one of two methods depending on the situation.

### A variant of the method to correctly determine the amount

In order to achieve better water suppression, a Fig. 1f pulse sequence (HSQC’) is occasionally used to obtain an HSQC spectrum. A τ_1_ - ^13^C 180° pulse - τ_1_ unit coupled with a gradient is inserted after the *t*_1_ period of a Fig. 1a pulse sequence. When the Fig. 1f pulse sequence is used, the volume of each peak of a curdlan is much smaller than 1 (Fig. 2d), the average being 0.538 (Table 1). The average volume of curdlan peaks for a Fig. 1f pulse sequence is even smaller than that for a Fig. 1a pulse sequence. This is because loss of magnetization through transverse relaxation during the inserted period is also greater for a curdlan than for a lignin dimer. As measures to this situation, a unit of τ_1_/4 - ^1^H 180° pulse - τ_1_/4 - ^13^C 180° pulse - τ_1_/4 - ^1^H 180° pulse - τ_1_/4 is inserted prior to the *t*_1_ period of a Fig. 1d pulse sequence to make a Fig. 1g pulse sequence. The total duration of the inserted unit is τ_1_. Loss of magnetization through transverse relaxation occurs during this unit for τ_1_. Here, we define γ for each peak as


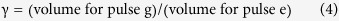


Then, the volume of each HSQC peak obtained with a Fig. 1f pulse sequence (HSQC’) is divided by γ ^2^ of each peak (Fig. 2e). In the case of a Fig. 1f pulse sequence, extra loss of magnetization occurs for a period of 2τ_1_. The volume for pulse g includes the effect of magnetization loss during the τ_1_ period. When the exponential-dependency of loss of magnetization on the period of relaxation is taken into account, it is regarded that a square of the volume for pulse g includes the effect of magnetization loss during the 2τ_1_ period. Therefore, division by γ ^2^ can compensate for the extra loss of magnetization during the 2τ_1_ period. Figure 2e clearly indicates that this method works well. All the volumes for a curdlan recovered, the average being 1.000 (Table 1). Thus, even when the Fig. 1f pulse sequence is used, correct quantitation can be achieved.

## Discussion

In our methods, the steady state ^13^C magnetization is utilized. Its longitudinal relaxation time is generally longer than that of ^1^H magnetization. Therefore, longer recycling delay time is required to ensure the recovery of the ^13^C magnetization, which results in the increase of experimental time to some extent. In a case of the present sample, the ^13^C longitudinal relaxation time was longer for a curdlan than a lignin model dimer, and the required recycling delay time was ca. 3.5 s which is five times as long as the ^13^C longitudinal relaxation time of a curdlan. The use of relaxation enhancing agents would be useful to reduce the experimental time, if necessary.

When intermolecular hydrogen bonds are formed, the apparent molecular size and rotational correlation time increase, which results in a fast transverse relaxation rate. Therefore, the presence of intermolecular hydrogen bonds also skews quantitation. Intermolecular hydrogen bonding is well known for cellulose and hemicelluloses, which are two of the three major components of woody biomass^13^. HSQC does not allow correct quantitation for these cases. However, the methods described here produce quantitative measures that are not affected by variations in the transverse relaxation rate. Therefore, this method is also valid for a system involving intermolecular hydrogen bonding, as is the case for woody biomass materials.

Quantitation with an HSQC spectrum is skewed not only by variation in the transverse relaxation rate among components of a mixture but also by variation in a one-bond C–H coupling constant, ^1^*J*_CH_. In order to maximize the efficiency of magnetization transfer during the INEPT and reverse INEPT periods, *τ* of the Fig. 1a pulse sequence is set to 1/(4∙^1^*J*_CH_). The ^1^*J*_CH_ values differ significantly between aliphatic and aromatic C–H bonds for components involved in woody biomass, the natural ^1^*J*_CH_ range being 115–220 Hz^7^. Therefore, when a certain *τ* value is set for HSQC, the efficiency of magnetization transfer differs for each component. This affects the volume of each HSQC correlation peak, which results in skewing of the quantitation. Again, however, the loss of magnetization during the INEPT and reverse INEPT periods due to mismatching between *τ* and 1/(4∙^1^*J*_CH_) for each component is automatically corrected with our method, when either HSQC/α^2^ or HSQC/β^2^ is calculated.

Finally, homonuclear ^1^H–^1^H couplings cause modulation during the INEPT and reverse INEPT periods, which results in the reduction of the volume of an HSQC correlation peak^7^. Our method can correct this reduction as well. The Q-HSQC method was successfully developed to correct the skewing of quantitation due to variation in ^1^*J*_CH_. Q-HSQC, however, cannot correct the skew caused by either homonuclear ^1^H–^1^H couplings or the variation in molecular size. In contrast, any loss of magnetization during the INEPT and reverse INEPT periods is corrected with our method, irrespective of its cause, to achieve correct quantitation. In this context, it should be added that the skew caused by the imperfection of each NMR pulse used in the INEPT and reverse INEPT periods is also corrected with our method.

The HSQC_0_ method was successfully developed for the correct quantitation^14^. Extrapolation using a series of HSQC spectra acquired with incremented repetition times (the time between the end of the first ^1^H excitation pulse to the beginning of data acquisition) is carried out in this method. This method utilizes long pulse sequences and thus suffers the loss of the signal intensity due to transverse relaxation. Therefore, it is difficult to apply this method to high molecular weight samples. Our method, the TAF quantitation method, utilizes the steady state ^13^C magnetization, as mentioned above. The polarization of ^13^C magnetization is smaller than that of ^1^H magnetization, ca. 1/4, which results in the low sensitivity of the signal originating from the steady state ^13^C magnetization. Although the use of the small polarization of ^13^C is an unpreferable point, it should be noticed that the unpreferable factor of 1/4 remains the same, without getting worse, even for high molecular weight samples. In addition, it should also be pointed out that the steady state ^13^C magnetization is free from the loss of the intensity due to transverse relaxation during the INEPT period of TROSY. Therefore, it is supposed that the TAF quantitation method is superior to the HSQC_0_ method for the application to high molecular weight samples.

In summary, the method described here, the TAF quantitation method, is tolerant of any factors that could skew quantitation: major variation in molecular size, intermolecular hydrogen bonding, variation in the ^1^*J*_CH_ value, presence of homonuclear ^1^H–^1^H couplings, and imperfections in NMR pulses. Moreover, the TAF method is applicable to high molecular weight samples. The developed TAF quantitation method is robust and generally applicable in a variety of different situations to assure correct quantitation, including in biomass plant cell wall NMR studies.

## Methods

### Sample Preparation

A lignin model dimer, guaiacylglycerol-*β*-guaiacyl ether, which models the most abundant linkage in lignin (Supplementary Fig. S1), was purchased from TCI, Japan (Tokyo). A curdlan, a high molecular weight linear polymer consisting of *β*-1,3-linked glucose residues (Supplementary Fig. S1), was purchased from Wako Pure Chemicals Industries Ltd (Osaka, Japan). The lignin dimer and curdlan were dissolved together in DMSO-d_6_. Initially, the concentrations of the lignin dimer and a curdlan were ca. 100 mM and 25 mg/ml, respectively. A one-dimensional ^1^H NMR spectrum was recorded. Then, on the basis of the area of each peak in the spectrum, the concentrations were further adjusted so that the concentration of the lignin dimer and that of a monomer unit of the curdlan were equal.

### NMR Spectroscopy and Manipulation of Data

NMR spectra were recorded using Bruker DRX600 and AVANCEIII700 spectrometers, each equipped with a cryoprobe. HSQC and TROSY spectra were recorded using pulse sequences a-g shown in Fig. 1 at 298 K. The spectrum obtained with each pulse sequence was recorded four times. The volume of the peak corresponding to each CH moiety was obtained, and its average and standard deviation were calculated for four independent experiments for each pulse sequence. The volumes or calibrated volumes were normalized so that the average of the volume for the peaks of the lignin dimer is 1. Data were processed and analysed with NMRPipe/NMRDraw^15^ and Sparky^16^. Pulse programs and parameters are available on request.

## Additional Information

**How to cite this article**: Okamura, H. *et al.* Accurate and molecular-size-tolerant NMR quantitation of diverse components in solution. *Sci. Rep.* **6**, 21742; doi: 10.1038/srep21742 (2016).

## Supplementary Material

Supplementary Information

## Figures and Tables

**Table 1:** The averages of the volumes of individual HSQC peaks for a lignin dimer and a curdlan.

	lignin	curdlan
HSQC	1.000 + /− 0.016	0.671 + /− 0.034
HSQC/α^2^	1.000 + /− 0.021	0.989 + /− 0.023
HSQC/β^2^	1.000 + /− 0.017	1.025 + /− 0.038
HSQC'	1.000 + /− 0.027	0.538 + /− 0.052
HSQC’/γ^2^	1.000 + /− 0.043	1.000 + /− 0.088

The average for the lignin dimer is normalized to 1. Numbers that follow + /− are standard deviations of individual HSQC peaks for a lignin dimer and a curdlan.
