# Supplementary material for: Accurate and molecular-size-tolerant NMR quantitation of diverse components in solution
Source: Sci Rep. 2016 Feb 17;6:21742. doi: 10.1038/srep21742 (PMC4756365; doi:10.1038/srep21742)
Supplement: Supplementary Information [file srep21742-s1.doc]

**Supplementary Information**

**Accurate and molecular-size-tolerant NMR quantitation of diverse components in solution**

**Hideyasu Okamura, Hiroshi Nishimura, Takashi Nagata, Takanori Kigawa, Takashi Watanabe & Masato Katahira**


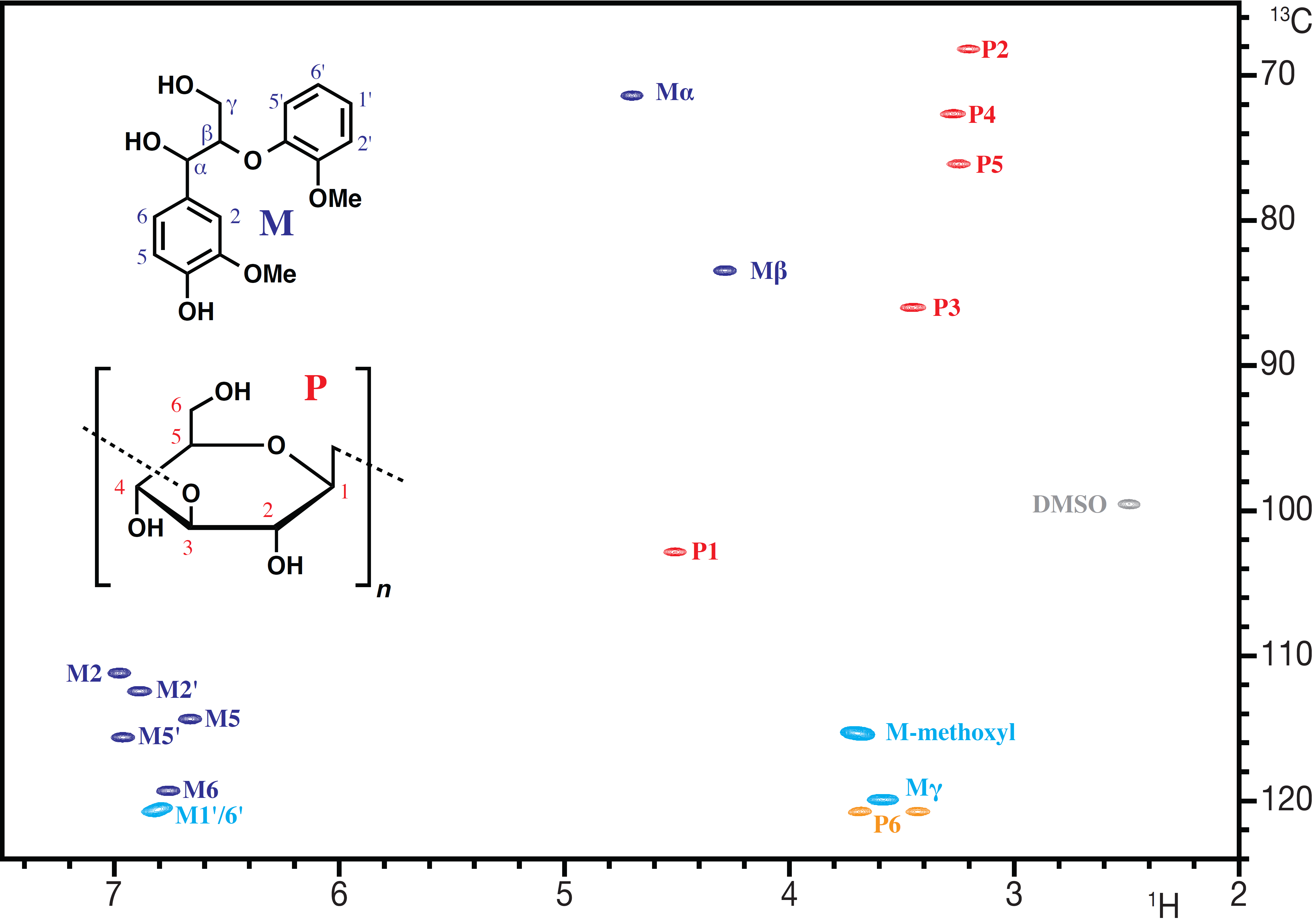


**Supplementary Figure S1**. **HSQC spectrum of a mixture of a lignin dimer (M) and the curdlan (P) with their molecular structures and assignments.** The spectrum was recorded with the Fig. 1A pulse sequence. The peaks of the lignin dimer are colored either dark or light blue, while those of the curdlan are colored either red or orange. Light blue and orange peaks are folded ones.
